# *In vitro* incubation of human sperm does not change DNA methylation

**DOI:** 10.1093/hropen/hoag079

**Published:** 2026-09-11

**Authors:** Emma Tykesson Kjøller, Marie-Charlotte Dumargne, Lars Roed Ingerslev, Eleonora Manitta, Yasmin Dehestani, Marte Saupstad, Alba Gonzalez-Franquesa, Atul Shahaji Deshmukh, Emil Andersen, Romain Barrès

**Affiliations:** Novo Nordisk Foundation Center for Basic Metabolic Research, Faculty of Health and Medical Sciences, University of Copenhagen, Copenhagen, Denmark; Institut de Pharmacologie Moléculaire et Cellulaire, CNRS, Inserm and Université Côte d‘Azur, Valbonne, France; Novo Nordisk Foundation Center for Basic Metabolic Research, Faculty of Health and Medical Sciences, University of Copenhagen, Copenhagen, Denmark; Novo Nordisk Foundation Center for Basic Metabolic Research, Faculty of Health and Medical Sciences, University of Copenhagen, Copenhagen, Denmark; Novo Nordisk Foundation Center for Basic Metabolic Research, Faculty of Health and Medical Sciences, University of Copenhagen, Copenhagen, Denmark; Novo Nordisk Foundation Center for Basic Metabolic Research, Faculty of Health and Medical Sciences, University of Copenhagen, Copenhagen, Denmark; Novo Nordisk Foundation Center for Basic Metabolic Research, Faculty of Health and Medical Sciences, University of Copenhagen, Copenhagen, Denmark; Novo Nordisk Foundation Center for Basic Metabolic Research, Faculty of Health and Medical Sciences, University of Copenhagen, Copenhagen, Denmark; Novo Nordisk Foundation Center for Basic Metabolic Research, Faculty of Health and Medical Sciences, University of Copenhagen, Copenhagen, Denmark; ExSeed Health ApS, Copenhagen, Denmark; Novo Nordisk Foundation Center for Basic Metabolic Research, Faculty of Health and Medical Sciences, University of Copenhagen, Copenhagen, Denmark; Institut de Pharmacologie Moléculaire et Cellulaire, CNRS, Inserm and Université Côte d‘Azur, Valbonne, France

**Keywords:** spermatozoa, DNA methylation, epigenetics, ART, bisulfite sequencing

## Abstract

**STUDY QUESTION:**

Is DNA methylation in mature spermatozoa affected by different culture media?

**SUMMARY ANSWER:**

Incubation of mature spermatozoa in hydrogen peroxide (H₂O₂) or palmitate for 24 h did not alter the DNA methylome.

**WHAT IS KNOWN ALREADY:**

Children conceived through ART face a higher risk of disorders linked to abnormal DNA methylation. As culture media can modulate DNA methylation in somatic cells, abnormal DNA methylation after ART may be caused by factors present in culture media during sperm incubation.

**STUDY DESIGN, SIZE, DURATION:**

Sperm from four donors underwent targeted shotgun proteomics analysis, focusing on protein size ranges that include epigenetic modifying enzymes. Additionally, sperm samples from 15 men were incubated for 24 h in either standard media, media with H₂O₂, or media containing palmitate, two media known to induce acute DNA methylation changes in somatic cells. DNA methylation profiles of these incubated sperm samples were then compared to those of pre-incubation sperm using Reduced Representation Bisulfite Sequencing (RRBS).

**PARTICIPANTS/MATERIALS, SETTING, METHODS:**

Sperm samples were obtained from 15 healthy Caucasian men aged 20–40 years. Participants using prescription medications daily or with a history of testicular disease were excluded. Motile sperm were isolated through the swim-up technique. Protein analysis was conducted via in-gel digestion followed by mass spectrometry. DNA methylation was evaluated using RRBS and validated for select genomic regions with targeted bisulfite pyrosequencing.

**MAIN RESULTS AND THE ROLE OF CHANCE:**

Proteomic analysis returned 1930 proteins, including 144 newly identified proteins. Consistent with previous studies, we confirmed the presence of DNA methyltransferase 1 (DNMT1), while *de novo* methyltransferases DNMT3A and DNMT3B were not detected. Incubation in control, H₂O₂, and palmitate media all led to decreased sperm motility, with the decline occurring the fastest under the H₂O₂ condition. Sperm DNA methylation remained unchanged across all three culture conditions.

**LARGE SCALE DATA:**

N/A

**LIMITATIONS, REASONS FOR CAUTION:**

RRBS analysis covers only a portion of the genome, so DNA methylation changes in unexamined regions cannot be excluded, particularly at repeated regions of the genome. Moreover, since this study tested only three culture media conditions, it remains possible that other media formulations could impact DNA methylation.

**WIDER IMPLICATIONS OF THE FINDINGS:**

This study indicates that the DNA methylome of mature human spermatozoa is stable despite the presence of DNA methylation machinery components. These results imply that DNA methylation changes in response to environmental stress are more likely to happen during earlier stages of spermatogenesis. While the various culture media used in ART are unlikely to alter the DNA methylation patterns of individual sperm cells, the possibility that certain sperm with specific methylation signatures are selectively favored cannot be ruled out.

**FUNDING:**

This work was supported by a Challenge Programme Grant from the Novo Nordisk Foundation (NNF18OC0033754) to the Gametic Epigenetics Consortium against Obesity (GECKO). The Novo Nordisk Foundation Center for Basic Metabolic Research is an independent research center at the University of Copenhagen, partially funded by an unrestricted donation from the Novo Nordisk Foundation (NNF18CC0034900). This work was supported by the French Government (National Research Agency, ANR) through the ‘Investments for the Future’ programs LABEX SIGNALIFE ANR-11-LABX-0028-01 and IDEX UCAJedi ANR-15-IDEX-01.

**DISCLOSURES:**

R.B. is a recipient of research funding granted by the Novo Nordisk Foundation. E.A. is a shareholder of ExSeed Health Ltd.

WHAT DOES THIS MEAN FOR PATIENTS? Children conceived through assisted reproduction, such as IVF, may have a slightly higher risk of certain health problems linked to changes in epigenetic marks—chemical signals placed onto the DNA molecules, which help control how an embryo develops.

Because these marks can be influenced when cells are grown in the laboratory, we studied whether IVF conditions could also affect the epigenetic marks of mature sperm. We exposed sperm from 15 individuals under conditions known to influence epigenetic marks in other cell types.

Although we found that sperm contain some of the molecules capable of modifying epigenetic marks, the exposure did not change the marks themselves. This suggests that mature sperm epigenetic marks are unlikely to be altered during assisted reproduction.

The increased occurrence of epigenetic disorders observed after assisted reproduction may instead be related to other factors, such as maternal health and/or the selection of sperm carrying pre-existing epigenetic abnormalities.

## Introduction

Infertility—defined as the failure to conceive after 1 year of regular, unprotected sex—impacts 15–20% of couples worldwide (World Health Organization, 2023). The widespread inability to conceive naturally has led to a significant rise in the use of ART (Kamphuis *et al.*, 2014). Although most ART-conceived children are healthy, their risk of imprinted disorders jumps by 4–9%, depending on the disease (Eroglu and Layman, 2012; Hattori *et al*., 2019). Moreover, some face greater chances of preterm birth, low birthweight, congenital malformations, and even perinatal mortality (Potiris *et al*., 2024). Untangling how much of these risks come from paternal infertility itself versus the ART procedures remains challenging.

Assisted reproduction procedures often involve semen preparation and incubation in embryo culture media, which can impact sperm DNA integrity (Pacchierotti and Spanò, 2015). Growing evidence suggests that the composition of culture media used in ART may influence offspring health, as different incubation conditions affect embryo quality and pregnancy success rates (Castillo *et al*., 2020; Coy *et al*., 2022). While some studies have linked culture media to variations in birth weight (Kleijkers *et al.*, 2016; Li *et al*., 2025), others have found no significant effects (De Vos *et al.*, 2015; Maas *et al.*, 2016). Animal research further shows that embryo culture environments can alter developmental programming (Watkins *et al.*, 2007; Chen *et al.*, 2019), increasing the risk of metabolic diseases, and reducing lifespan (Watkins *et al.*, 2007; Chen *et al.*, 2014). Together, these findings highlight that culture media may play a role in shaping disease risks for ART-conceived offspring.

The mechanisms by which ART media may impact the health of the offspring are largely unknown. Changes to the epigenetic make up of spermatozoa may be involved. Sperm DNA methylation indeed controls embryo development (Aston *et al.*, 2015; Jenkins *et al.*, 2016) and is influenced by environmental and lifestyle factors such as diet, weight loss, and exercise (Donkin *et al.*, 2016; Jönsson *et al.*, 2025). Thus, it is plausible that sperm incubation during ART could affect DNA methylation profiles of spermatozoa, which in turn would impact embryo development and offspring health. While the sperm DNA methylome of the same individual can be remodeled within a week post bariatric surgery (Donkin *et al.*, 2016), it is unknown whether these epigenetic changes occur in terminally differentiated spermatozoa. The presence of epigenetic machinery components in mature sperm would support that DNA methylation can be remodeled in mature sperm, potentially during ART.

The actual presence of DNA methylation-modifying enzymes in mature spermatozoa remains uncertain. Although immunolocalization studies have detected DNA methyltransferases (DNMT1, DNMT3a, and DNMT3b) (Marques *et al.*, 2011), and *in situ* hybridization and qRT-PCR have identified ten-eleven translocation (TET) enzymes (TET1, TET2, and TET3) (Ni *et al.*, 2016), other research comparing RNA-seq and sperm proteomic data suggests that only DNMT1 is present in mature sperm (Castillo *et al.*, 2018). Some studies, however, have been unable to detect any of these enzymes at all (Shen *et al.*, 2019; Luo *et al.*, 2022).

In this study, we set out to uncover which DNA methylation-related proteins are present in human mature sperm and to find out if DNA methylation can be reshaped when sperm are exposed to different culture environments. We discovered multiple key epigenetic regulators inside mature spermatozoa, yet surprisingly, DNA methylation patterns remained largely unchanged—even after treatment with an oxidative stress inducer (hydrogen peroxide (H₂O₂)) or the fatty acid palmitate. These results suggest that DNA methylation in sperm is remarkably stable, even under conditions commonly used in ART, shedding new light on sperm epigenetic resilience.

## Materials and methods

### Study cohort

Twenty participants were recruited with the following inclusion criteria: age 20–40 years, Caucasian descent, and body mass index 20–28 kg/m^2^ (Table 1). Exclusion criteria were daily use of prescription medicine and previous serious conditions, including conditions associated with reproductive organs. Two participants were excluded due to total sperm concentration <15 million spermatozoa per milliliter. All remaining 18 participants were further studied in all assays, except 3 who were excluded from the Reduced Representation Bisulfite Sequencing (RRBS) analysis due to technical errors during sequencing. The study was approved by the Regional Committee on Health Research Ethics, reference: H-17041722. Informed consent was acquired from all participants.

**Table 1.** Clinical parameters of participants.

Clinical parameters	*N* = 15
Age [years]	25.1 ± 1.7
Height [cm]	183 ± 7.8
Weight [kg]	77.7 ± 8.2
BMI [kg/m^2^]	23.2 ± 1.7
SBP [mmHg]	130.3 ± 14.8
DBP [mmHg]	79.5 ± 10.9
Alcohol/week [units]	7.4 ± 4.3
Current smokers [n]	1
Volume [ml]	3.9 ± 1.2
Concentration [million sperm/ml]	100.69 ± 101.07
Total sperm count [million sperm/ejaculate]	340.84 ± 210.43
Total motility [%]	36.2 ± 10.9
Progressive motile cells [%]	26.76 ± 9.3
Non-progressive motile cells [%]	9.4 ± 4.6
Immotile cells [%]	63.8 ± 11.0

BMI, body mass index; SBP, systolic blood pressure; DBP, diastolic blood pressure.

Data are mean ± SD.

### Semen sample collection

Single ejaculate samples were collected directly into sterile polypropylene containers (Sarstedt, Nümbrecht, Germany) from each participant after 3-5 days of abstinence. Quickly after ejaculation, samples were placed at 37°C for 30 min for liquefaction. Basic sperm parameters were inspected by phase contrast microscopy according to WHO guidelines (WHO, 2010) at room temperature (22°C). The total amount of the ejaculate was determined by wide-bore volumetric pipetting. Sperm samples with <15 million spermatozoa/ml were excluded.

### Swim-up procedure

Motile spermatozoa were isolated from non-motile spermatozoa and somatic cells by swim-up. Briefly, 0.5 ml of semen was carefully overlaid with 2 ml swim-up media in 14 ml round-bottom tubes (Thermo Fisher Scientific, Waltham, MA, USA). Swim-up media contains Earle’s Balanced Salt Solution (Sigma-Aldrich, Darmstadt, Germany), 3.2 mg/ml Human Serum Albumin (Sigma-Aldrich), and 5.96 mg/ml Hepes (Sigma-Aldrich). The tubes were then incubated at 37°C at a 45° angle for 2 h. After incubation, each upper fraction containing the motile sperm cells was pooled per ejaculate and homogenized. Spermatozoal fractions before and after swim-up were analyzed, and the presence of monomorphic malformation was assessed. Spermatozoa were counted at room temperature (22°C), and the presence of somatic cells was determined.

### Shotgun proteome analysis of sperm samples

Shotgun proteomics was used on a pool of four sperm samples. As the lysis method results in the discovery of different proteins (Shen *et al.*, 2019; Luo *et al.*, 2022), we adapted two protocols for a total of four lysis conditions (Zhou *et al.*, 2015; Carapito *et al.*, 2017). Protocol 1 was used for proteome analysis of intact sperm cells; 200 μl buffer containing 7 M urea, 2 M thiourea, 65 mM dithiothreitol, and 1% of v/v protease inhibitor cocktail was added to a pellet of 50 million spermatozoa (Zhou *et al.*, 2015). The sample was homogenized with a syringe at 4°C, followed by 1 h of vigorous stirring at 4°C. One round of sonication (5 × 20 s) was performed at 4°C. Protocols 2-4 were used to test both Triton X-100 soluble fractions (supernatant), the pellet after centrifugation and a mix of both, respectively (Carapito *et al.*, 2017). A volume of 300 μl 1% Triton X-100 was then added to the pellet, and the solution was vigorously stirred for 1 h at 4°C, followed by centrifugation for 15 min at 10 000*g* at 4°C. The supernatant was removed and kept. The pellet was washed in MilliQ water (Merck Millipore, Burlington, MA, USA), and 2% SDS was added. The supernatant was then precipitated by adding 300 μl methanol and 150 μl chloroform for a total of 750 μl. The solution was mixed, followed by a 2 min centrifugation at 10 000*g*. The upper phase was discarded, and 225 μl methanol was added. The sample was inverted twice and centrifuged for 15 min at 15 000*g*. The supernatant was again discarded, and the pellet was resuspended in 50 μl 2% SDS. Protein concentrations were measured. After protein isolation of all samples, an in-gel-digestion was made, using Colloidal Blue Staining Kit (Sigma-Aldrich). Gel fractions were cut into two different fractions for each sample. Upper fractions contain proteins in the range of 150–250 kDa for the detection of TET1, TET2, TET3, and DNMT1. Lower fractions contain proteins in the range of 75–125 kDa for the detection of DNMT3A and DNMT3B. Fractions were sliced into small 1 mm cubes and transferred to 2 ml microtubes for washing before incubation overnight at 37°C with trypsin solution and ABC buffer. Peptide extraction was done by centrifugation. Thirty-five microliters of 30% acetonitrile (ACN)/3% trifluoroacetic acid (TFA) solution was used, followed by a 20 min incubation at room temperature in a shaker. The supernatant was transferred into a new 1.5 ml tube; 100 μl of 30% ACN/3% TFA solution was added followed by a 20 min incubation at room temperature in a shaker. The supernatant was transferred to the same tube as the other supernatant. One hundred microliters of 80% ACN/0.5% acetic acid was added to cover gels followed by a 20 min incubation at room temperature in a shaker. The supernatant was transferred to the same tube as the other supernatant; 100 μl 100% ACN buffer was added, followed by a 20 min incubation at room temperature in a shaker. The supernatant was transferred to the same tube as the other supernatant. Samples were concentrated using a SpeedVac instrument (Thermo Fisher Scientific) at 45–60°C for 45 min (V-AL mode) for evaporation to a total volume of ∼150 μl per tube. These were then acidified by adding 100 μl 0.1% TFA/0.5% acetic acid solution. Samples were spun down for 2 min at maximum speed, and supernatants transferred into StageTips (Empore, Oxford, PA, USA). Proteins were eluted, and solvent was then evaporated using a SpeedVac for ∼25 min, prior to peptide resuspension. Samples were then kept at −20°C until Mass Spectrometry. Peptides were injected into an Easy nano-flow HPLC system (Thermo Fisher Scientific) coupled to an LTQ Q-Exactive Orbitrap Mass Spectrometer via a nanoelectronspray source (Thermo Fisher Scientific). Samples were then loaded into a 15 cm C18-particle packed column (inner diameter 75 μm, 1.8 μm beads, Dr. Maisch GmbH, Ammerbuch, Germany) with Buffer A (0.5% formic acid) and separated over a 45-min non-linear gradient from 2–30% Buffer B (80% acetonitrile, 0.5% formic acid) at a flow rate of 250 nl/min. Mass spectra were acquired in a data-dependent manner, automatically switching between MS and MS/MS using a Top 10 method. Raw MS spectra were analyzed using MaxQuant software (v1.5.5.1). Peaks were matched to the Uniprot Human FASTA database, including a common contaminants database, including forward and reverse sequences using Andromeda search engine. Perseus software (v1.6.5.0) was used for downstream analysis of LFQ intensities.

### Sperm incubation

The upper fraction of each swim-up was diluted to 8 ml in swim-up media and further divided into four fractions. One fraction was directly transferred to a 2 ml DNA-LoBind tube (Eppendorf, Hamburg, Germany) and was used as the pre-incubation sample for reference. We analyzed sperm under the microscope to confirm the absence of somatic cell contamination. Their purity was later further analyzed by looking at DNA methylation levels of imprinted genes, which confirm the efficiency of our method to purify pure spermatozoa. The three remaining were split into a 12-well plate (Costar, Corning Incorporated, Corning, NY, USA), with each row for one condition, control media, 0.5 mM Palmitate (Sigma-Aldrich), and 1 mM H₂O₂ (Sigma-Aldrich), and incubated at 37°C for 24 h. Motility and viability were evaluated at 1, 2, 4, and 24 h whenever the experimental conditions permitted. After incubation, samples were transferred to 2 ml DNA-LoBind tubes (Eppendorf). Samples were spun down and washed once in PBS before the addition of 650 µl RLT Plus (Qiagen, Hilden, Germany) with 1% β-mercaptoethanol. Samples were mixed and stored at −80°C until DNA extraction.

### DNA isolation

For DNA methylation analysis, genomic DNA was extracted from the sperm cells by a method adapted from Wu *et al.* (2015). Briefly, cell lysis was achieved by adding one 5 mm stainless steel bead to each tube (Qiagen) to the sperm cells. The tubes were then placed in a TissueLyser II (Qiagen) for 2 min at 25 Hz. Excessive foam production was eliminated by a quick spin. DNA was isolated and purified with the AllPrep DNA/RNA/miRNA Universal Kit (Qiagen). DNA concentrations were measured by NanoDrop 2000 Spectrophotometer (ThermoFisher Scientific) and Qubit® Fluorometer dsDNA HS Assay Kit (Life Technologies).

### RRBS library preparation

Genome-wide DNA methylation analysis at a single-base resolution was performed using RRBS as previously described (Andersen *et al.*, 2019). Briefly, 100 ng of genomic DNA of each condition was incubated with Mspl enzyme (EpiTect®, Qiagen) in order to fragment DNA at CCGG positions to enrich for CpG regions. Bisulfite conversion was carried out using the EpiTect® Bisulfite Conversion kit (Qiagen), according to the manufacturer’s instructions. DNA was then PCR-amplified and ligated to TruSeq sequencing adapters (Illumina). Library preparation was performed using the NuGEN Ovation® RRBS Methyl-Seq kit (Tecan Genomics, Redwood City, CA, USA) as instructed by the manufacturer. Library integrity was assessed by the Qubit® Fluorometer dsDNA HS Assay Kit (Thermo Fisher Scientific) and the Bioanalyzer High-sensitivity DNA chip (Agilent Technologies, Santa Clara, CA, USA). Libraries were then pooled and sequenced on a NextSeq^TM^ 500 System (Illumina, San Diego, CA, USA) by 75 bp single-end sequencing and processed by CASAVA 1.8.2. On average, 25.3 million reads/sample were aligned to the reference genome (hg38) and 214 131 CpGs had sufficient coverage in every sample.

### Data processing and RRBS data analysis

The raw sequencing data were processed as previously described (Andersen *et al.*, 2019). Briefly, RRBS reads were pre-processed using Trim Galore to remove adaptor sequences, the bases added during RRBS library preparation, and the bases with a Phred score <30. Alignment to the hg38 reference genome assembly, deduplication, and summarization was performed by Bismark with the -rrbs flag set and default parameters. Methylation status of every cytosine in a CpG context was extracted using ‘bismark_methylation_extractor’. CpG methylation calls were summarized using the bismark2bedgraph script, which is part of the Bismark software suite. Samples with <10 million aligned reads were observed to have different global levels of methylation and were excluded from further analysis. For each CpG, observations from both strands were added together to form an overall methylation level per CpG, and only CpGs covered by eight or more reads in every sample were used for testing for differential methylation. Bigwig files were obtained with bedGraphToBigWig (Kent *et al*., 2010), and methylation values were visualized with Integrative Genome Viewer (Robinson *et al*., 2011). Coverage files generated by Bismark were imported into R, and figures were generated using ggplot2. CpGs overlapping NCBI dbSNP Build 151 (Sherry *et al*., 2001) were excluded. Each CpG overlapping a promoter or a coding sequence was annotated with the gene ID, and each CpG was overlapped with a CpG cluster, where a cluster is defined as a stretch of DNA with no more than 100 bp between CpGs.

Testing for differential methylation was performed by different methods, first using edgeR either on single CpGs or using counts aggregated within clusters, where each cluster was defined by scanning along the genome and grouping CpGs no more than 100 bp apart, or using counts aggregated within promoters (defined as 3000 bp upstream and 1000 bp downstream of TSS) and gene bodies. Only regions with eight or more observations were tested. Lastly, differential methylation was also tested between groups using the DMR-seq package (Korthauer *et al*., 2019). Originally designed for analyzing whole-genome bisulfite sequencing data, the default parameters for region construction and smoothing can be adjusted to fit the enzymatic sequencing data resulting from RRBS. Parameters were adjusted as follows: cutoff = 0.05, minInSpan = 10, maxGapSmooth = 4000, while maxPerms and bpSpan were kept as default. Adjusting these parameters affected the sizes of differentially methylated regions (DMRs) identified, but the procedure still provided valid inference.

### Targeted bisulfite pyrosequencing

Bisulfite-pyrosequencing was performed to validate RRBS findings; 1 µg of genomic DNA was bisulfite-converted using the EZ DNA Methylation-Lightning KIT (Zymo Research, Irvine, CA, USA) according to the manufacturer’s recommendations, and 30 ng of bisulfite-converted DNA per sample was amplified with the PyroMark PCR kit (Qiagen, Germany) following the manufacturer’s instructions. Contamination was assessed by agarose gel electrophoresis. Pure amplicons were sequenced on a PyroMark Q48 Autoprep instrument (Qiagen). Pyrosequencing primers were designed using the PyroMark Assay Design Software. Specific primers are listed in Supplementary Table S1. Data were analyzed with the provided PyroMark Q48 Autoprep software.

### Bioinformatics and statistical analysis in R

All bioinformatics analyses and statistical evaluations were conducted using R version 4.2.1 (R Core Team, 2022) and relevant Bioconductor packages. Pathway and gene ontology enrichment analyses were performed using the clusterProfiler package (Yu *et al*., 2012). Data visualization and figure generation were carried out using ggplot2 (Wickham, 2016) and associated tidyverse packages. Figures were compiled and arranged in Adobe Illustrator (Adobe Inc., San Jose, CA, USA) for final publication-quality presentation.

## Results

### DNMT and TET enzymes are largely absent in mature human spermatozoa

To investigate whether DNMTs and TET enzymes are present in mature spermatozoa, we employed four different protein isolation techniques combined with targeted shotgun proteomics. We focused on two gel fractions: one containing proteins sized 75–125 kDa, corresponding to DNMT3A and DNMT3B, and another ranging from 150 to 250 kDa, corresponding to TET1, TET2, TET3, and DNMT1 (Fig. 1A). In total, we identified 1956 protein families, representing 2151 unique proteins matched to the Uniprot Human FASTA database (Supplementary File S1). Among these, 144 proteins had not been previously detected in human sperm, including bromodomain-containing protein 4 (BRD4), a key regulator of chromatin. Most proteins (93.3%) overlapped with those reported in earlier human sperm proteomic studies (Castillo *et al.*, 2018; Shen *et al.*, 2019; Luo *et al.*, 2022; Pini *et al.*, 2025) (Fig. 1B, Supplementary Table S2). Importantly, while we confirmed the presence of DNMT1, we did not detect DNMT3A, DNMT3B, or any TET enzymes, suggesting that proteins responsible for *de novo* methylation or active demethylation are absent in mature spermatozoa.

**Figure 1.**
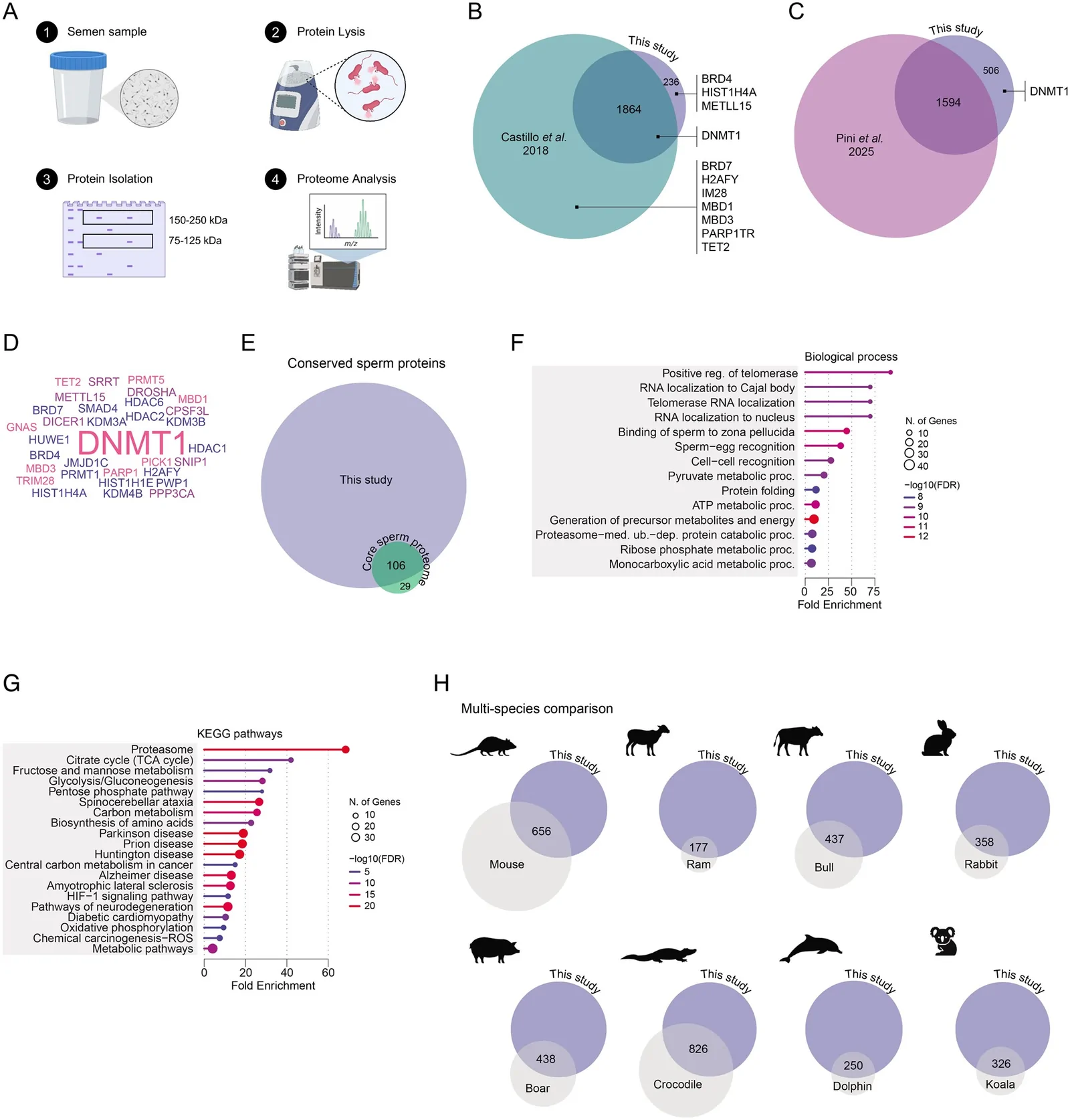
**Comprehensive sperm proteome analysis across species reveals conservation, functional enrichment, and epigenetic regulators**. (**A**) Schematic overview of the targeted sperm proteome analysis performed in this study. (**B** and **C**) Venn diagrams illustrating the number of sperm proteins identified in this study compared to previously reported datasets (Castillo *et al*., 2018; Pini *et al*., 2025), highlighting overlapping and unique protein sets (BRD4, DNMT1, and TET2). (**D**) Summary of conserved sperm proteins with roles in epigenetic regulation, RNA localization, and chromatin remodeling, reinforcing their potential involvement in sperm function and fertilization competence. (**E–G**) KEGG pathway and biological process enrichment analysis of conserved sperm proteins, indicating functional categories such as monocarboxylic acid metabolism, pyruvate metabolism, ATP metabolism, and sperm–egg recognition or revealing significant enrichment in metabolic pathways, oxidative phosphorylation, and pathways associated with neurodegenerative diseases. (**H**) Cross-species comparison of identified sperm proteins in various species (e.g. mouse, ram, boar, bull, rabbit, dolphin, koala, crocodile), revealing a conserved core proteome as well as human-specific differences.

### Sperm DNA methylation patterns remain stable after 24 h *in vitro* incubation

In non-dividing cells, H₂O₂ and palmitate have been shown to cause rapid DNA methylation changes (Barrès *et al.*, 2009, 2012; Hall *et al*., 2014; Maples *et al*., 2015). To assess whether DNA methylation patterns in mature spermatozoa can be altered by different culture conditions, we isolated motile sperm and incubated them for 24 h in either control media or media supplemented with H₂O₂ or palmitate (Fig. 2A). Sperm DNA methylation profiles were investigated on samples from 15 participants (Supplementary Fig. S1C; Supplementary Table S3). Semen parameters were within the WHO reference values for semen volume (>1.4 ml), sperm concentration (>16 million/ml), and total sperm count (>39 million), while nine participants had below-normal total motility (<42%, WHO). Both motility and viability were significantly reduced under stress conditions, particularly with H₂O₂ treatment (Fig. 2B; Supplementary Fig. S2A and B), indicating compromised sperm function.

**Figure 2.**
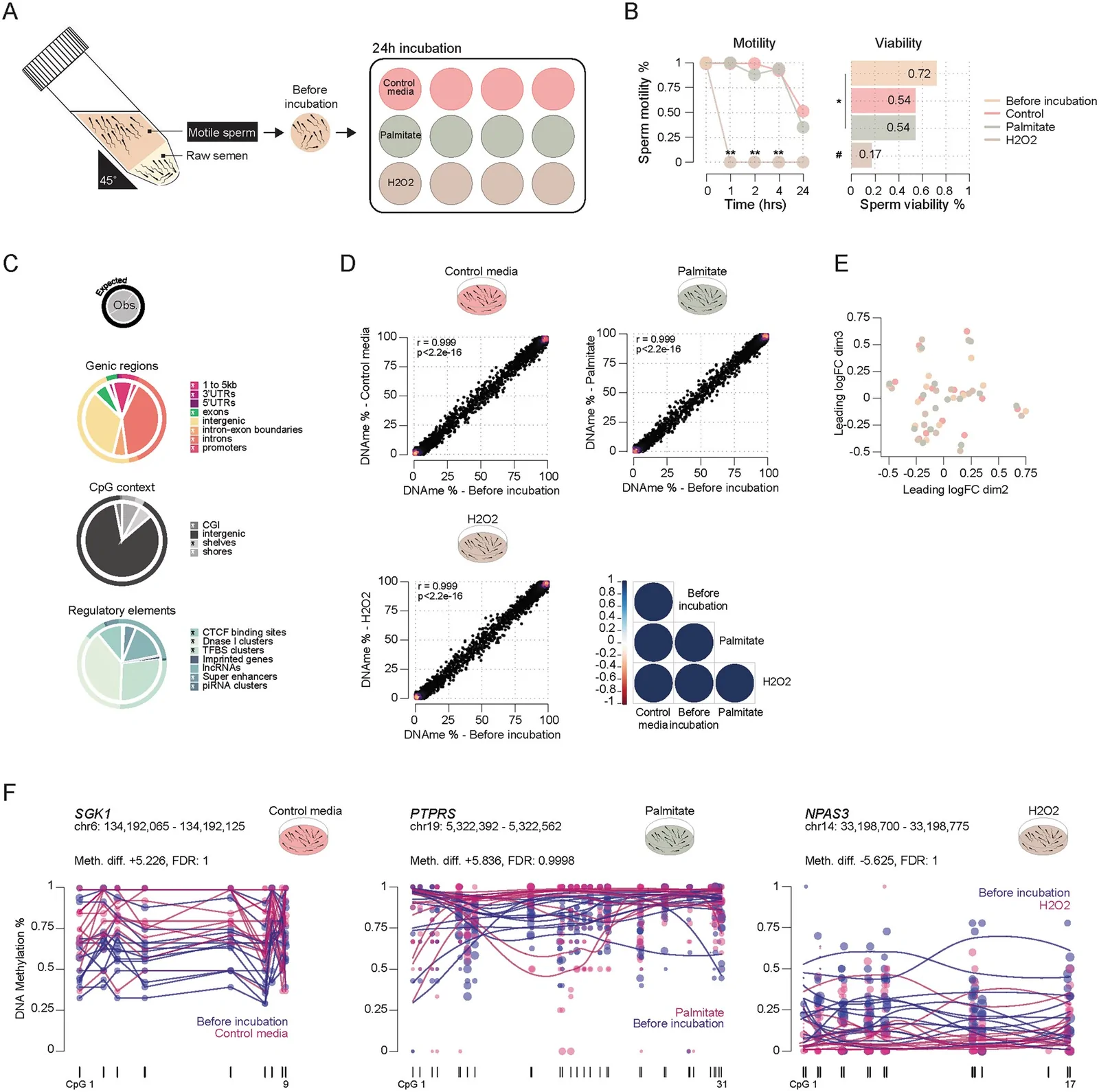
**Culture conditions do not alter sperm DNA methylation profiles**. Schematic overview of the experiment for the isolation of motile sperm from raw semen (**A**) and the 24 h incubation of mature motile spermatozoa in control media or media supplemented with H₂O₂ or palmitate. (**B**) Sperm motility and viability measurements over time and by condition. Left: motility over a 24-h period. Right: viability percentages across treatments. Asterisks indicate statistical significance compared to control. Mean values with SEM of differences between conditions were calculated using two-way ANOVA and Dunnett’s multiple comparisons test. **P ≤ 0.05 versus Control (n = 5). (**C**) Genomic context analysis of differentially methylated CpG sites, showing their distribution across genic regions (e.g. exons, introns, promoters) and regulatory elements (e.g. CTCF binding sites, DNase I clusters, super enhancers). Asterisks indicate statistical enrichment compared to Reduced Representation Bisulfite Sequencing (RRBS) background. (**D**) Correlation plots comparing baseline DNA methylation levels (before incubation) to post-treatment levels (H₂O₂, palmitate, control media), showing no differences (Pearson’s *r* = 0.999, *P* < 2.2 × 10^−16^). (**E**) Multi-Dimensional Scaling (MDS) of DNA methylation levels, showing no separation of samples based on treatment and incubation status. (**F**) DNA methylation levels across representative CpG sites in gene regions of interest (e.g. neuronal PAS domain protein 3 (NPAS3), protein tyrosine phosphatase receptor type S (PTPRS), and serum glucocorticoid regulated kinase 1 (SGK1)) under different conditions: before incubation, control media, H₂O₂, and palmitate treatment. Each panel shows methylation percentage (*y*-axis) across individual CpGs (*x*-axis) within selected genomic coordinates, with methylation difference (meth. diff.) and false discovery rate (FDR) values indicated.

To evaluate genome-wide DNA methylation, RRBS was conducted, achieving an average sequencing depth of 25.3 million aligned reads per sample (Supplementary Table S3). Consistent with the targeted nature of RRBS, coverage across CpG-rich regions reached a median of ∼100×, ensuring robust methylation calling and reliable downstream analysis (Supplementary Fig. S1D). The DNA methylation coverage exhibited the characteristic RRBS enrichment pattern, with preferential coverage of CpG-dense regions—including promoters, exons, introns, CpG islands, shores, and shelves—while intergenic and imprinted regions were comparatively underrepresented (Fig. 2C). This bimodal distribution of DNA methylation at CpG sites was consistent across all experimental conditions (Fig. 2D; Supplementary Fig. S2A and B). Multi-Dimensional Scaling analysis identified Participant 10 as an outlier with distinct DNA methylation patterns compared to other individuals (Supplementary Fig. S2C). Given that participant 10 was the sole smoker in the cohort, this sample was excluded from subsequent analyses. After removal of this outlier, no distinct clustering by experimental condition was observed (Fig. 2E). Analysis of sperm DNA methylation profiles revealed a high degree of concordance between pre- and post-incubation samples, indicating overall stability of the sperm DNA methylome following incubation.

Comparative analysis of DNA methylation profiles before and after incubation identified 19 DMRs in cells cultured with control media, 14 DMRs following palmitate exposure, and 25 DMRs under H₂O₂ treatment (Fig. 2F; Supplementary File S2). However, none of these DMRs remained statistically significant after adjustment for multiple testing using a false discovery rate threshold of 0.05. Among the initially identified DMRs, the majority exhibited hypomethylation, representing 69%, 70%, and 71% of DMRs in control, palmitate, and H₂O₂ conditions, respectively. Representative loci showing modest methylation changes included an increase of 5.23% in methylation near SGK1 (serum glucocorticoid regulated kinase 1) in control media, a decrease of 5.62% near NPAS3 (neuronal PAS domain protein 3) under H₂O₂ treatment, and an increase of 5.83% near PTPRS (protein tyrosine phosphatase receptor type S) with palmitate exposure (Fig. 2F; Supplementary File S2). Across all identified DMRs, the magnitude of DNA methylation changes between pre- and post-incubation remained below the 10% threshold. The limited number of DMRs and the modest methylation differences observed cannot be attributed to insufficient coverage or low sequencing depth, as RRBS sequencing covered a total of 214 131 CpG sites with an average sequencing depth of 25.3 million reads per sample (Supplementary Table S3). These findings indicate that DNA methylation patterns are largely stable in mature spermatozoa under the conditions tested.

### Imprinted and transposable regions are not affected by 24 h incubation in cell culture media

Altered methylation at imprinted regions has been linked to the higher incidence of imprinting disorders observed in children conceived through ART (Eroglu and Layman, 2012). Considering this, we aimed to evaluate the stability of DNA methylation at imprinted loci in sperm following incubation. Specifically, we analyzed six imprinted regions—*H19*, *KCNQ1*, *MEG3*, *DIRAS3*, *SNRPN*, and *GRB10*—covered by RRBS, all of which have consistently been reported to exhibit significant methylation changes in ART-conceived offspring (*H19*: Barberet *et al.*, 2021a; Loke *et al.*, 2015; Sakian *et al.*, 2015; *MEG3*: Nelissen *et al.*, 2013; Jiang *et al.*, 2021; *KCNQ1*: Gomes *et al.*, 2009; Rancourt *et al.*, 2012; Chen *et al.*, 2018). These loci have also been suggested to undergo differential methylation during ART procedures (Lou *et al.*, 2019; Barberet *et al*., 2021b).

To determine whether oxidative or lipid stress affects epigenetic regulation at imprinted loci, we focused on these imprinted regions across treatment conditions. Methylation stability across these loci was confirmed (Fig. 3A–F). Indeed, average CpG methylation across H19, KCNQ1, MEG3, SNRPN, DIRAS3, and GRB10 remained stable regardless of incubation with H₂O₂, palmitate, or control media. This finding was consistently observed in both RRBS data (Fig. 3A–F) and pyrosequencing assays (Supplementary Fig. S3A–C). Quantitative comparison (Fig. 3G) confirmed that no significant methylation changes occurred at these loci following treatment, suggesting that imprinting marks are resilient to short-term environmental stressors in human sperm.

**Figure 3.**
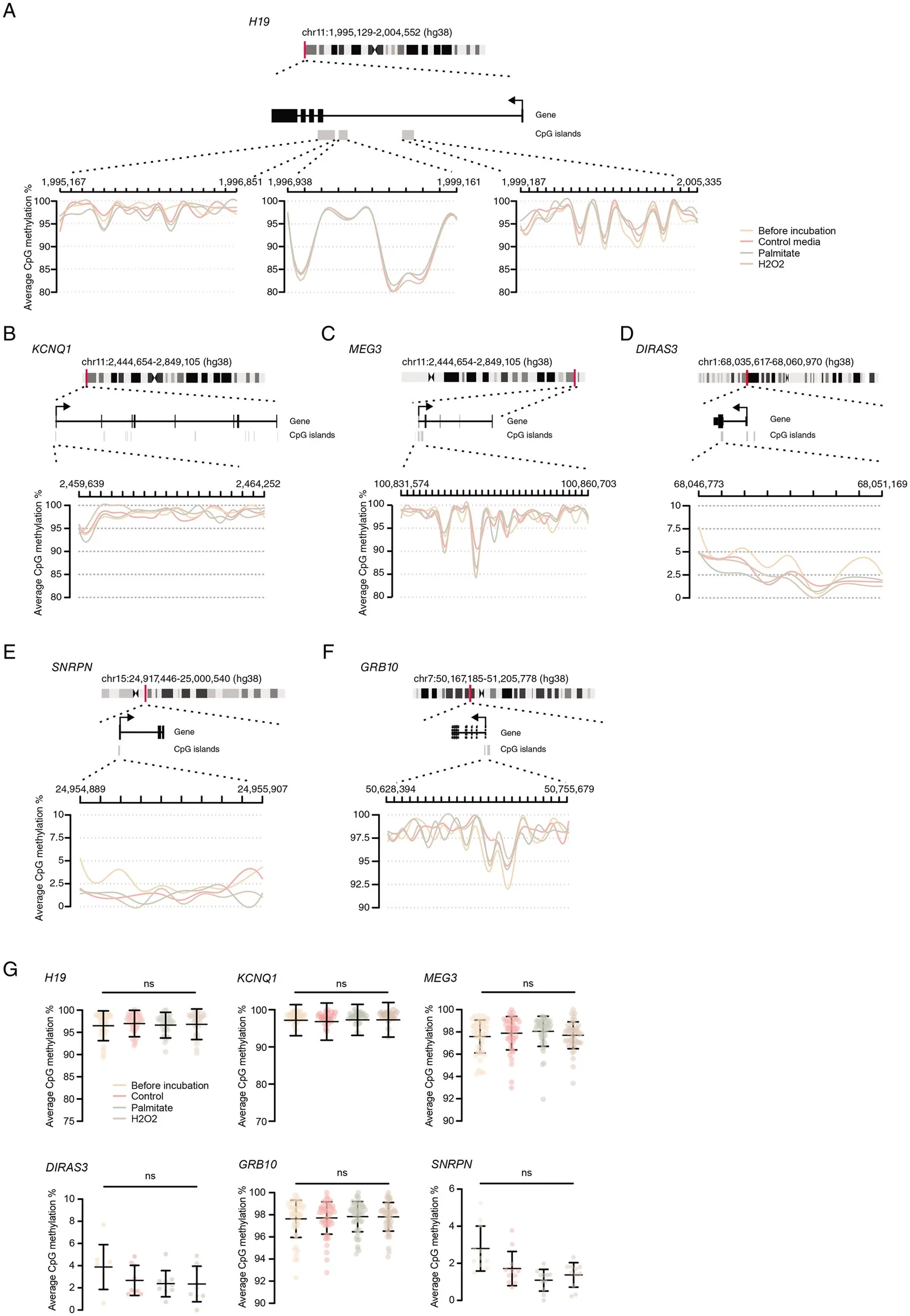
**DNA methylation at imprinted gene loci remains stable across stress conditions**. (**A**–**F**) Average CpG methylation levels across imprinted gene regions: H19, KCNQ1, MEG3, SNRPN, DIRAS3, and GRB10. Each plot shows methylation levels across genomic intervals covering CpG islands and associated gene bodies, with genomic coordinates based on hg38. Samples include sperm before incubation, and after exposure to control media, H₂O₂, or palmitate. (**G**) Box plots summarizing average CpG methylation for each locus across conditions. No significant differences were observed across treatments, indicating robust methylation stability at these imprinted loci (ns: not significant).

### Stability of DNA methylation at transposable elements under stress conditions

Another genomic feature is transposable elements (TEs), which make up nearly half of our DNA. Because young TEs can potentially jump around and disrupt genetic information, they are kept under tight control by DNA methylation to prevent their activation and safeguard genomic stability. A phylogenetic snapshot of TE families reveals the rich diversity and evolutionary journey of these repetitive sequences (Fig. 4A). While RRBS is not specifically designed to target TEs, our dataset included sufficient coverage across a subset of TE regions across all major classes to confidently assess potential methylation changes. It is important to note that by design, RRBS preferentially targets CpG-rich regions, including promoters, CpG islands, and regions enriched in older TE sequences (Gu *et al*., 2011). These older TEs, such as ancient long interspersed nuclear elements and short interspersed nuclear elements (SINEs), are typically no longer transcriptionally active and are considered epigenetically inert, as they are not actively regulated by DNA methylation in the germline or somatic cells (Slotkin and Martienssen, 2007). Consequently, while RRBS provides robust coverage of gene promoters and regulatory elements, it also captures a substantial proportion of methylation data from these evolutionarily ancient, inactive TEs (Fig. 4B). TE distribution across the genome was non-uniform (Fig. 4C), with chromosome 19 exhibiting the highest density of TEs. This observation is consistent with the fact that chromosome 19 is the most gene-rich and GC-rich chromosome in the human genome–genomic features known to favor the historical accumulation of SINE elements such as Alu. Across all TE classes and treatment groups, DNA methylation levels remained uniformly high and stable (Fig. 4D). Stratification of TEs by evolutionary age further confirmed this stability, with no significant differences observed between young and old elements under any condition (Fig. 4E). Genome browser views of representative regions on Chromosomes 19 and 17 (Fig. 4F and G) reinforced these findings, showing consistent methylation patterns at annotated TEs regardless of treatment. These results indicate that DNA methylation at TEs in human sperm is remarkably stable and not perturbed by short-term oxidative or metabolic stress.

**Figure 4.**
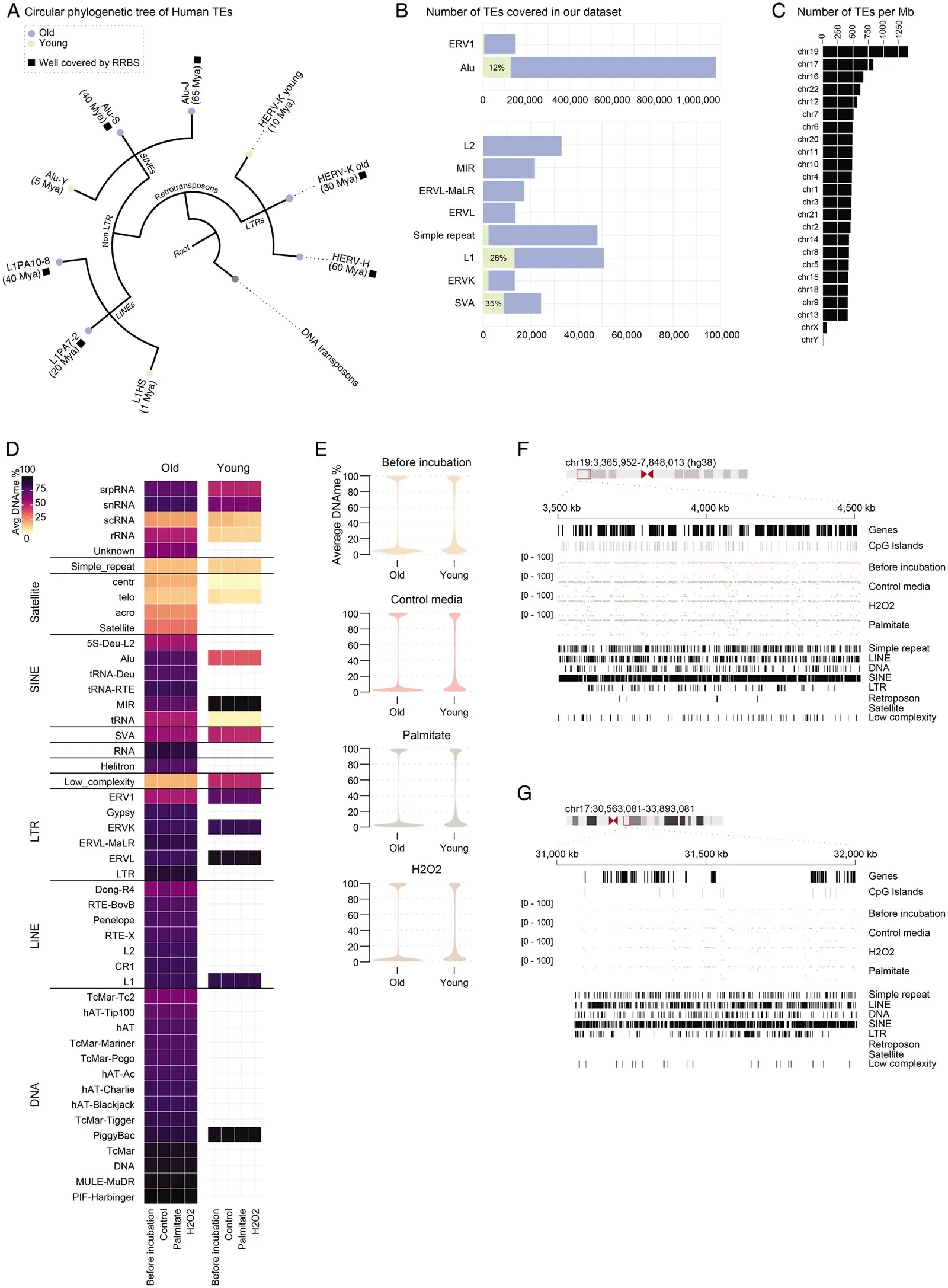
**DNA methylation at transposable elements is stable across stress conditions**. (**A**) Circular phylogenetic tree of major human transposable element (TE) families, categorized by class (short interspersed nuclear element (SINE), long interspersed nuclear element (LINE), long terminal repeats (LTR), DNA transposons) and annotated by evolutionary age. (**B**) Distribution of TEs across human chromosomes, shown as number of TEs per megabase (Mb), highlighting the variability in TE density. (**C**) Proportion of TEs captured by RRBS in this dataset, stratified by TE class. (**D**) Average DNA methylation levels across TE classes (e.g. LINE, SINE, LTR, DNA transposons) under each condition: before incubation, control media, H₂O₂, and palmitate. *Old* and *Young* refer to the age of TE (**E**). Comparison of methylation levels between evolutionarily young and old TEs across all conditions shows consistently high methylation with no significant differences. (**F** and **G**) Genome browser views of representative genomic regions (on chr19 and chr17), showing methylation tracks and annotated TEs. No detectable changes in TE methylation are observed across experimental conditions.

Overall, these findings provide compelling evidence that DNA methylation is highly stable in mature human spermatozoa, even under *in vitro* oxidative or metabolic stress. This suggests that the sperm DNA methylation landscape, particularly at imprinted loci, is well-preserved and is unlikely to be directly affected by gamete manipulation during ART. Therefore, any epigenetic alterations seen in ART-conceived offspring are more likely to occur after fertilization, rather than resulting from changes in the sperm methylome itself.

## Discussion

In this study, we set out to investigate whether DNA methylation-modifying enzymes are present in mature spermatozoa and to assess the potential for DNA methylation remodeling in these cells following incubation in culture media known to induce methylation changes in other cell types. Our analysis confirmed the presence of DNMT1 in mature spermatozoa but found no evidence for the *de novo* methyltransferases DNMT3A and DNMT3B, suggesting that active DNA methylation remodeling is unlikely. This conclusion was further supported by our observation that treatment with H₂O₂ or palmitate did not result in any detectable changes in DNA methylation patterns, either globally or at specific genomic loci.

Using mass spectrometry, we confirmed the presence of DNMT1 in mature spermatozoa, aligning with previous proteomic studies, but did not detect other DNA methylation-related enzymes, such as TET1, TET3, DNMT3A, or DNMT3B, which have been reported by others (Wang *et al.*, 2013; Zhou *et al.*, 2015; Vandenbrouck *et al.*, 2016; Carapito *et al.*, 2017). Previous studies employing qRT-PCR and immunofluorescence have suggested the presence of DNMT1, DNMT3A, and DNMT3B in mature spermatozoa and throughout development, with DNMT1 and DNMT3B detected in the sperm head and DNMT3A in the tail (Uysal *et al.*, 2016). Additionally, mRNA for *TET1-3* has been reported at various stages of spermatogenesis, including in mature spermatozoa (Ni *et al.*, 2016). The discrepancies among studies may reflect differences in detection sensitivity (e.g. antibody-based assays versus mass spectrometry) or the detection of remnant, untranslated transcripts. Furthermore, our inability to identify certain proteins could stem from their low abundance, shared peptide sequences, degradation, or biases in protein isolation. Our mass spectrometry analysis identified 144 additional proteins in mature spermatozoa compared to prior reports (Castillo *et al.*, 2018; Shen *et al.*, 2019; Luo *et al.*, 2022), highlighting the depth of our dataset. The spermatozoa being translationally inert, detection of mRNA of genes involved in epigenetic editing may not be functionally relevant. Moreover, the sole mass-spectrometry detection of DNMT1 without assessment of its enzymatic activity does not imply functional relevance. Nonetheless, the absence of *de novo* methyltransferases is likely a key factor underlying the remarkable stability of DNA methylation in mature spermatozoa.

The absence of detectable DNA methylation changes following sperm incubation might be attributed to the limited (∼1%) genome coverage of RRBS. Furthermore, RRBS predominantly targets GC-rich regions, which are thought to be more resilient to environmental perturbations compared to regions with intermediate methylation (Weber *et al.*, 2007). To gain a more comprehensive view of DNA methylation stability in mature spermatozoa, future studies employing long-read sequencing or whole-genome bisulfite sequencing may be warranted.

We also found that both palmitate and H₂O₂ exposure significantly reduced sperm motility and viability, with motility declining rapidly compared to controls. Given that palmitate and H₂O₂ can induce oxidative stress and DNA damage (Barzilai and Yamamoto, 2004), it is plausible that the oxidative insult overwhelmed the epigenetic machinery, precluding the occurrence or detection of acute DNA methylation changes during incubation.

Demonstrating the absence of acute DNA methylation changes following sperm incubation is inherently challenging. However, if DNA methylation is indeed stably maintained in ejaculated sperm, the increased incidence of imprinting disorders observed in ART-conceived offspring may instead be caused by maternal factors and/or a bias in selecting spermatozoa that already possess altered methylation profiles at imprinted loci. This highlights the importance of refining sperm selection techniques in ART to prioritize the identification and isolation of sperm populations with optimal DNA methylation profiles, as the handling procedure itself is unlikely to directly alter the sperm methylome (Aston *et al.*, 2015; Jenkins *et al.*, 2016). Current sperm selection techniques—including swim-up, density gradient centrifugation, and microfluidic chips—primarily focus on physical parameters such as motility, morphology, and DNA fragmentation (Wong *et al.*, 1986; Vaughan and Sakkas, 2019; Baldini *et al.*, 2021). However, innovative strategies that enable the selection of epigenetically healthy spermatozoa may help to further optimize ART outcomes (Aston *et al.*, 2015; Jenkins *et al.*, 2016). By combining physical and epigenetic criteria in sperm selection, ART practices could enhance the likelihood of successful fertilization and support the development of healthy offspring.

In conclusion, our findings provide evidence that DNA methylation in mature spermatozoa remains remarkably stable, even under conditions that might induce methylation changes in other cell types. While we confirmed the presence of DNMT1, its enzymatic activity was not assessed, and the absence of *de novo* methyltransferases DNMT3A and DNMT3B further supports that methylation patterns in mature sperm are largely set and resistant to change. Although it is inherently challenging to definitively demonstrate the complete absence of DNA methylation alterations with RRBS, our data strongly suggest that the elevated risk of imprinting disorders observed in ART-conceived offspring is more likely to arise from maternal factors and/or the preferential selection of spermatozoa carrying pre-existing methylation anomalies, rather than from acute methylation changes induced by ART procedures themselves. These findings underscore the importance of considering both genetic and epigenetic integrity in ART practices to ensure the best possible outcomes for offspring health.

## Supplementary Material

hoag079_Supplementary_Data

## Data Availability

The data underlying this article are available in the article and in its online supplementary material.
