# Supplementary material for: In vitro incubation of human sperm does not change DNA methylation
Source: Hum Reprod Open. 2026 Sep 11;2026(4):hoag079. doi: 10.1093/hropen/hoag079 (PMC13682945; doi:10.1093/hropen/hoag079)
Supplement: Supplementary file 1 [file hoag079_supplementary_data.zip › HRO-26-0661 - Supplementary Figures Final.docx]

**Supplementary Figures**

**In-vitro incubation of human sperm does not change DNA methylation**

Emma Tykesson Kjøller^1Ϯ^, Marie-Charlotte Dumargne^2Ϯ^, Lars Roed Ingerslev^1^, Eleonora Manitta^1^, Yasmin Dehestani^1^, Marte Saupstad^1^, Alba Gonzalez-Franquesa^1^, Atul Shahaji Deshmukh^1^, Emil Andersen^1,3*^ & Romain Barrès^1,2*^

**Supplementary Figure S1 - Influence of culture incubation sperm viability.** Differences in (A) sperm viability following a four-hour incubation, and (B) sperm motility among participants. (C) Overview of the study design, including samples excluded due to low sperm concentration or sequencing failure. (D) The ridgeline plot depicts the read coverage (in X-fold depth) across CpG-rich regions targeted by RRBS in all samples. The median coverage was approximately 100X, with a broad distribution reflecting variability across targeted genomic loci. The high coverage across these regions ensures accurate methylation profiling for downstream analysis.

**Supplementary Figure S2 - Distribution and differential methylation of sperm CpG sites.** Bimodal distribution of sperm DNA methylation levels. The number of individual CpG sites with a minimum read depth of 10 is shown, categorized by their methylation status (A) prior to incubation, and (B) after incubation in control media or media supplemented with H₂O₂ or palmitate. (C) Multi-Dimensional Scaling (MDS) analysis of DNA methylation levels reveals a distinct separation of participant 10 from the other samples. (D) Volcano plots illustrate differences in DNA methylation, with each point representing an analysed region. Coloured points denote regions with an adjusted p-value < 0.05, while grey points indicate non-significant changes. Positive β-values correspond to hypermethylation, and negative β-values indicate hypomethylation in each condition compared to the control (pre-incubation).

**Supplementary Figure S3 - Pyrosequencing of some imprinted genes.** (A-C) DNA methylation levels at imprinted regions were assessed by pyrosequencing, consisting in 3 CpGs sites in the H19 locus, 5 CpGs sites in the MEG3 locus, and 6 CpGs sites in the KCNQ1 locus. No significant differences were observed across different treatments. Data are presented as the mean ± SEM of differences, determined by Tukey’s multiple comparisons test.
